# Chronic ankle instability modifies proximal lower extremity biomechanics during sports maneuvers that may increase the risk of ACL injury: A systematic review

**DOI:** 10.3389/fphys.2022.1036267

**Published:** 2022-10-18

**Authors:** Yue Xu, Bin Song, Anghan Ming, Congda Zhang, Guoxin Ni

**Affiliations:** ^1^ Department of Sports Medicine, Sun Yat-sen Memorial Hospital, Guangzhou, Guangdong, China; ^2^ School of Sports Medicine and Rehabilitation, Beijing Sport University, Beijing, China

**Keywords:** ankle instability, lower extremity, joint biomechanics, sports activities, anterior cruciate ligament

## Abstract

The biomechanical changes in the lower extremity caused by chronic ankle instability (CAI) are not restricted to the ankle joint, but also affect the proximal joints, increasing the risk of joint injury. This study aimed to systematically review the research on CAI and lower extremity angle and movements during side-cutting, stop jumping, and landing tasks, to provide a systematic and basic theoretical basis for preventing lower extremity injury. Literature published from exception to April 2022 were searched in the PubMed, Web of Science, and SPORTDiscus databases using the keywords of “chronic ankle instability,” “side-cut,” “stop jump,” and “landing.” Only studies that compared participants with chronic ankle instability with healthy participants and assessed lower extremity kinetics or kinematics during side-cutting, stop jumping, or landing were included. The risk of bias assessment was conducted using a modified version of the Newcastle-Ottawa checklist. After title, abstract, and full text screening, 32 studies were included and the average score of the quality evaluation was 7 points (range 6–8). Among them five studies were related to the side-cut task, three studies were the stop-jump task, and twenty-four studies were related to landing. Although the results of many studies are inconsistent, participants with CAI exhibit altered lower extremity proximal joint movement strategies during side cut, stop jump, and landings, however, such alterations may increase the risk of anterior cruciate ligament injury.

## 1 Introduction

Ankle sprains are one of the most prevalent sports injuries, with 49.3% of ankle sprains occurring during sports (Fong et al., 2007). Ankle sprains, however, are frequently dismissed as minor ailments, with only around half of patients seeking professional help after their first sprain (Hubbard-Turner, 2019). Due to a lack of care and incorrect management, around 40% of patients will develop to chronic ankle instability (CAI) after an initial ankle injury (Doherty et al., 2016). CAI is indicated by recurrent sprains, instances of ankle joint giving way, pain, swelling, and impaired function (Hertel and Corbett, 2019). The person with CAI has a higher risk of recurring sprains (Gribble et al., 2016). Approximately 68%–78% of individuals with CAI develop ankle osteoarthritis as a result of impaired balance and neuromuscular control of the lower extremity during movement (Moisan et al., 2017), which decreases the health-related quality of life and sports participation (Arnold et al., 2011).

Any impact on the ankle joint will affect the knee and hip joints since the lower extremities are a chain of movements. During ankle sprains, the mechanoreceptors in the joint capsule are damaged, and the impulse flow from the mechanoreceptors to the central nervous system is disrupted, causing problems with joint position and motion perception, as well as posture and gait reflexes (Wyke, 1967). As a result, a cascade of adaptive reactions is triggered, leading to alterations in movement patterns (Bullock-Saxton,1994). According to the lower extremity kinematic chain theory, the person with CAI demonstrates proximal joint kinematic modifications during sports, which are designed to compensate for the ankle joint’s instability and functional impairment (Koshino et al., 2014).

Side cutting, stopping and landing are common sports movements, and they are also high-risk movements for anterior cruciate ligament (ACL) injury (Pflum et al., 2004; Chappell et al., 2007; Lin et al., 2009). Side cutting, which involves a change of direction and is a multi-plane movement, is common in court sports and also necessitates more complicated joint control for the lower limb than motions that occur in daily life (i.e. walking, running) (Koshino et al., 2016). While the stop jump task is a sport-related functional activity with a high risk of lower-limb injuries because the lower limb joints are required to endure the high impact energy exerted in a very short amount of time. The landing is typical of specific sports activity (Kim et al., 2018). Those sports maneuvers are more similar to real-life sports actions. Several studies comparing lower extremity biomechanical and neuromuscular control measures in CAI participants with healthy controls have been published, with some findings indicating that CAI participants had greater ankle dorsiflexion, knee flexion, and hip flexion (Caulfield and Garrett, 2002; Jeon et al., 2020), and prolonged peroneus longus latency during the landing phase of a single-leg drop (Simpson et al., 2019a). Terada et al. (2014a) demonstrated that participants with CAI demonstrated less knee flexion at peak anterior tibial shear force (ATSF) compared to the controls during stop jump. Altered lower extremity biomechanics due to CAI may make it much easier for patients to sustain non-contact ACL injuries (Theisen and Day, 2019). However, because our understanding of the complex pathological manifestations of CAI is still limited and many studies based on similar measurements have produced inconsistent results, there is still a necessity to better understand the mechanisms underlying recurrent sprains and limited sports participation in CAI. This study intends to use a systematic review to summarize, evaluate, and analyze the literature on sports biomechanics research on participants with CAI during side cuts, stops jump, and landing.

## 2 Methods

### 2.1 Search strategy

By April 2022, a literature search was conducted without regard to geographies, and publishing kinds. The databases searched included PubMed, Web of Science and SPORTDiscus. The computer search was supplemented with manual searches of the reference lists of all retrieved studies, review articles, and conference abstracts with the Related Articles tool. A Boolean logic method is used to write all database searches: (chronic ankle instability OR ankle instability OR functional ankle instability OR mechanical ankle instability) AND (lower limb OR lower extremity OR hip OR knee OR ankle) AND (kinematic OR kinetics OR biomechanics) AND: 1) side cut: (side cut OR side-cut OR side cutting OR side-cutting OR cut OR cutting), 2) stop jump: (stop jump OR stop-jump OR stop jumping OR stop-jumping), 3) landing: (land OR landing OR jump land OR jump landing OR jump-land OR jump-landing OR drop-vertical jump OR single-leg landing OR single-leg land OR jump OR jumping).

### 2.2 Inclusion and exclusion criteria

Two independent reviewers (BS and AHM) assessed titles and abstracts based on the inclusion criteria to identify eligible papers that would be subjected to full-text review. If the following criteria were met, studies were considered for full-text review: 1) published English language articles, 2) compared a CAI group to a healthy control group of participants, 3) main outcome measures were lower limb three joints angles and moments during side cut, stop jump, and landing, 4) study types were randomized controlled trials (RCTs) and retrospective comparative studies (including cohort or case-control studies), 5) if a study incorporated a therapy, it was only included if the control and CAI groups were compared before the intervention. Studies were excluded if the following criteria were met:1) non-English articles, 2) not compared with healthy controls, 3) main outcome measures were muscle activity, joint stiffness, energy dissipation, muscle energy production, ground reaction force, 4) animal experimental research, editorials, letters to the editor, review papers, case reports, and commentaries.

### 2.3 Risk of bias assessment

When disagreements arose, the same two independent authors (YX and GXN) assessed the study’s quality and discussed it together to reach a consensus. If the two authors were unable to reach an agreement, the final product was examined by a third author (CDZ). The Cochrane risk of bias tool was used to assess the methodological quality of RCTs (Higgins and Green, 2008). The modified Newcastle-Ottawa scale was used to assess the methodological quality of retrospective research (Taggart et al., 2001). The modified Newcastle-Ottawa scale has three components: patient selection, research group comparability, and outcome evaluation. Except for RCTs, each study was given a score between 0 and 9. Studies that received six or more points were deemed to be of good quality. The Centre for Evidence-Based Medicine in Oxford, United Kingdom, assessed studies based on the level of evidence they supplied (Howick et al. 2022).

### 2.4 Data extraction and analysis

Two of the authors independently gathered and summarized data from the included research (YX and GXN). The goal and quality of the study, participant characteristics, inclusion criteria, intervention protocols, and outcome variables were all assessed during the review process. The type of study, number and gender of participants, test site, test technique, foot condition, time frame, assessment parameters, assessment plane, lower extremity three-joint angle, and lower extremity three-joint moment were among the data retrieved. Any disagreement was resolved by the adjudicating senior authors (CDZ). No data pooling or meta-analysis was done since the included studies lacked homogeneity in terms of research methods, task site (flat or inverted), foot state (barefoot or shod), and CAI definition.

## 3 Result

### 3.1 Search results

**FIGURE 1:**
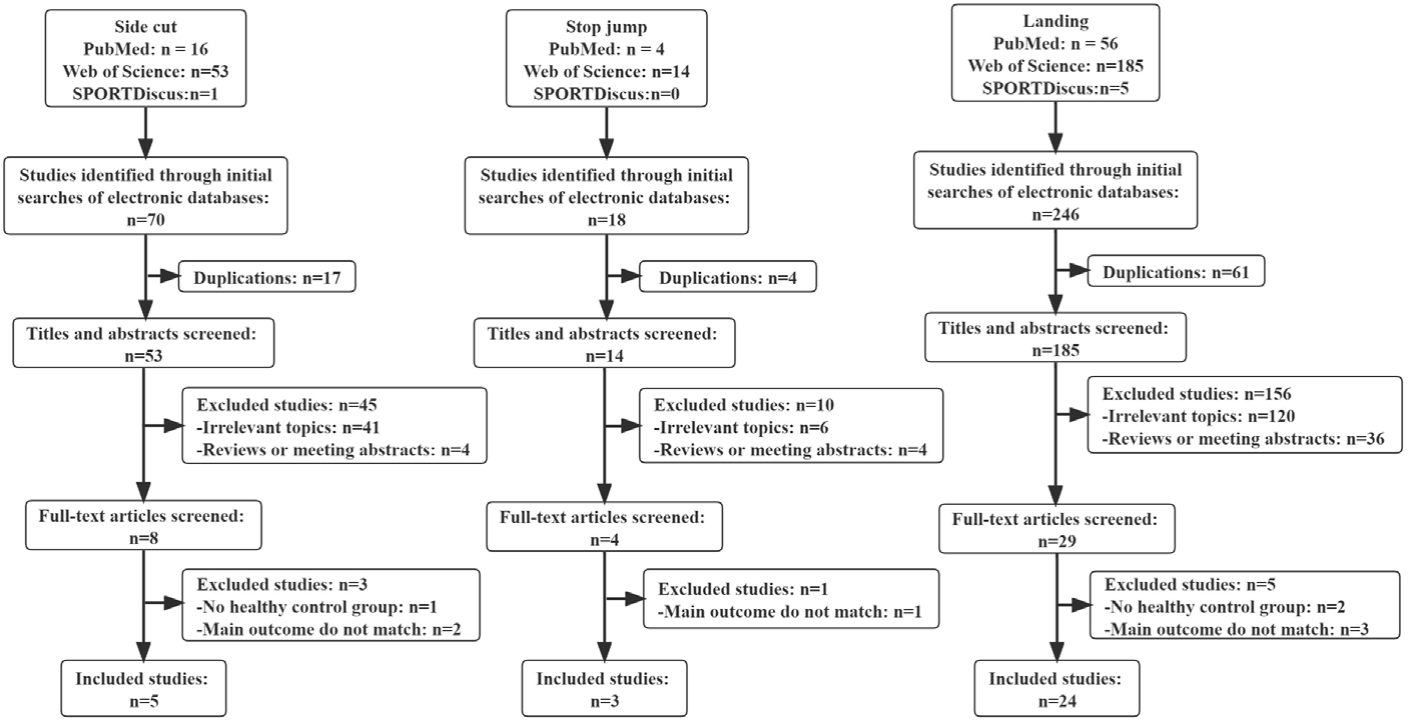
Flow chart of literature search and screening.

A total of 70 articles related to side-cutting were initially searched in electronic databases, 53 articles remained after removal of duplicate articles, eight articles remained after the title and abstract review, and after full-text review, one article was excluded due to non-inclusion of healthy controls, and two articles were excluded due to no main observations given, and five articles were finally included. A total of 18 articles related to stop jump were initially searched, 14 articles remained after removal of duplicate articles, four articles remained after title and abstract review, and after full-text review, one article was excluded due to main outcome did not match, and three articles were finally included. A total of 246 articles related to landing were initially searched, 185 articles remained after removing duplicate articles, 29 articles remained after title and abstract review, and after full-text review, two articles were excluded due to not including healthy controls, and three articles were excluded due to not giving the main observation index, and 24 articles were finally included. For study selection, there was 90% agreement between the two authors. Figure 1 depicts a flow diagram of the study selection process, as recommended by PRISMA.

### 3.2 Characteristics of eligible studies

A total of 32 articles were included after searching for three different sports maneuvers, without randomized controlled studies, all of which were retrospective, and the characteristics of the included literature are shown in Table 1. In five publications, the test technique was side-cutting; all literature did a 45° side-cutting task, and shoe-wearing in two articles, the rest were not specified. The test method for the stop jump included three articles, all of which were tested with shoes on. The test procedure involves landing with 24 articles, a shoe-wear test of eight articles, and a barefoot test of six articles, the rest not stated. The platform height range was 15–70cm, with flat landing surfaces in 21 articles, slanted ground in four, and unstable terrain in one. Because certain studies did not give particular means, standard deviations, or mean differences in the text, this review does not provide specific data for this research.

**TABLE 1:** Basic characteristics of the included studies.

Authors	Level of evidence	Design	Patients, no.		Matching^a^	Test method	Quality score
			CAI (M: F)	Control (M: F)			
Caulfield and Garrett, (2002)	—	NA	14 (14:0)	10 (10:0)	1,5	Single leg jump landing	7
Delahunt et al. (2006)	—	NA	24 (15:9)	24 (16:8)	3,4	Single leg jump landing	7
Gribble and Robinson, (2009)	4	CC	19 (10:9)	19 (10:9)	All	Double-leg take-off jump with a single-limb landing	8
Gribble and Robinson, (2010)	—	NA	19 (10:9)	19 (10:9)	All	Double-leg take-off jump with a single-limb landing	8
Lin et al. (2011)	4	CL	15 (6:9)	15 (7:8)	2,5	Vertical stop jump	7
Kipp and Palmieri-Smith, (2012)	—	NA	11 (5:6)	11 (5:6)	1,2,3,4,5	Double-leg take-off jump with a single-limb landing	6
Zhang et al. (2012)	—	NA	10	10	2,3,4	Double-leg take-off jump with a single-limb landing	7
Kipp and Palmieri-Smith, (2013)	—	NA	11 (5:6)	11 (5:6)	1,2,3,4,5	Double-leg take-off jump with a single-limb landing and 90°cut	6
Terada et al. (2014a)	4	CC	19 (10:9)	19 (10:9)	All	Vertical stop jump	8
Terada et al. (2014b)	4	CC	19 (10:9)	19 (10:9)	1,2,3,4,5	Vertical stop jump	7
Koshino et al. (2014)	4	CS	12 (10:2)	12 (10:2)	All	Forward jump and 45° crossover cut	8
Gehring et al. (2014)	—	NA	FAI + MAI: 19	18	NA	Double-leg drop–vertical-jump	6
			FAI: 9				
De Ridder et al. (2015)	—	NA	28 (10:18)	28 (10:18)	1,2,3,4,5	Double-leg take-off jump with a single-limb landing	7
Koshino et al. (2016)	4	CC	10 (9:1)	10 (9:1)	All	Forward jump and 45° crossover cut	8
Wright et al. (2016)	4	CS	23 (12:11)	23 (12:11)	1,2,3,4,6	Single leg jump landing	7
Son et al. (2017)	4	CL	22 (12:8)	22 (12:8)	1,3,4,5,6	Double-leg take-off jump with a single-limb landing and 90°cut	7
Fuerst et al. (2018)	4	CS	18 (8:10)	18 (8:10)	1,2,3,4	45°sidestep-cutting after a straight run	7
Herb et al. (2018)	4	CL	24	23	1,3,4,5,6	Double-leg drop–vertical-jump	7
Kim et al. (2018)	—	NA	100 (54:46)	100 (54:46)	All	Double-leg take-off jump with a single-limb landing and 90°cut	8
Li et al. (2018)	—	NA	21 (0:21)	21 (0:21)	All	Single leg landing	8
Kunugi et al. (2018)	4	CS	15 (15:0)	15 (15:0)	1,2,3,4	Double-leg take-off jump 45 °anterolateral with a single-limb landing and vertical-jump	7
McCann et al. (2019)	4	CC	25	25	2,3,4	Double-leg take-off jump with a single-limb landing	7
Kim et al. (2019)	—	NA	100 (54:46)	100 (54:46)	All	Double-leg take-off jump with a single-limb landing and 90°cut	8
Hopkins et al. (2019)	4	DL	200 (104:96)	100 (54:46)	5	Double-leg take-off jump with a single-limb landing and 90°cut	6
Lin et al. (2019)	—	NA	10 (8:2)	10 (8:2)	All	Single leg jump landing	8
Simpson et al. (2019b)	—	NA	15	15	1,3,4	Single leg landing	6
Moisan et al. (2020)	4	CC	32	31	1,2,3,4	Single leg jump landing	6
Jeon et al. (2020)	—	NA	MAI:10 (10:0)	10 (10:0)	1,2,3,4,6	Double-leg take-off jump with a single-limb landing	6
			FIA:10 (10:0)				
Simpson et al. (2020a)	3	C	15 (7:8)	15 (7:8)	1,2,3,4,6	Forward jump and 45° crossover cut	6
Simpson et al. (2020b)	—	NA	15 (7:8)	15 (7:8)	1,2,3,4,6	Forward jump and 45° crossover cut	6
Watabe et al. (2021)	4	CS	12 (12:0)	12 (12:0)	1,2,3,4,6	Single leg landing	7
Watanabe et al. (2022)	—	NA	11 (7:4)	11 (7:4)	All	Single leg landing	8

^a^ Comparability variables: 1 = gender; 2 = age; 3 = hight; 4 = weight; 5 = sports level; 6 = dominance side.

M, male; F, female; NA, data not available; C, Cohort Study; CC, Case-control study; CL, Control laboratory research; CS, Cross-sectional studies; DL, Descriptive laboratory studies.

### 3.3 Quality assessment

The mean score after scoring using the modified Newcastle-Ottawa scale (Taggart et al., 2001) was 7 (range 6–8, see Supplementary Table S1), with 97% agreement between the two reviewers.

### 3.4 Studies related to side cut

Only one of the five trials instructed participants to do a 45° sidestep-cutting maneuver following a straight approach run (Fuerst et al., 2018), whereas the other four asked them to do a forward jump and then a 45° crossover cut and run (Koshino et al., 2014; Koshino et al., 2016; Simpson et al., 2020b; Simpson et al., 2020a). Table 2 shows summaries of studies on side-cut. In the task which asked subjects to perform a forward jump and then a 45° crossover cut and ran, the CAI group exhibited significantly greater hip abduction (approximately 4.04°) from the 200 ms pre-IC (pre-initial contact) to 45% of the stance phase than the control group (Koshino et al., 2014), and greater hip flexion approximately 5.51° from 6% to 50% of the stance phase (Koshino et al., 2014), besides, Simpson et al. (2020a) found no differences between the groups in hip movements. In addition, the CAI group had considerably more knee flexion from 35 to 64% and 69%–87% of the stance phase than the control group, and the mean differences across groups were 7.63° and 9.54°, respectively (Koshino et al., 2014). In contrast, one study found no variations in knee angle between groups (Koshino et al., 2016). Only one study discovered that the CAI group had a lower knee abduction moment from 52% to 75% of the stance phase (difference = 0.27 ± 0.03 Nm/kg) (Simpson et al., 2020a). Almost the majority of the studies considered found differences in ankle angle or movement between the CAI group and healthy controls; only one research revealed no differences (Koshino et al., 2014). The CAI group exhibited significantly greater ankle inversion than the control group from 200 to 165 ms pre-IC (roughly 7.7°) and from 78 to 100% of the stance phase (roughly 6.4°) (Koshino et al., 2016). The CAI group had significantly greater ankle internal rotation from 35 to 54% of the stance phase compared to controls, with a mean difference of 6.62 ± 0.10° (Simpson et al., 2020b). The CAI group had a higher ankle plantar-flexion moment from 3% to 16% of the stance phase (difference = 0.22 ± 0.08 Nm/kg) and a lower ankle-eversion moment from 39% to 80% of the stance phase (difference = 0.13 ± 0.02 Nm/kg) than the control group (Simpson et al., 2020a). The findings of the task, which required individuals to complete a 45° sidestep-cutting maneuver after a straight approach run, only showed that the CAI group had smaller maximum inversion angles than the control group (Fuerst et al., 2018).

**TABLE 2:** Summary of articles related to side cut.

Authors	Task	Foot condition	Planes	Time frame	Variables	Hip	Knee	Ankle
Koshino et al. (2014)	Forward jump and 45° crossover cut	Shod	F + S + T	(-)200 m to	lower extremity joints angles	↑FL (6–50%SP)	↑FL (35–64%, 69–87%SP)	NS
						↑ABD ((-)200 m to 45%SP)		
Koshino et al. (2016)	Forward jump and 45° crossover cut	Shod	F + S + T	(-)200 m to	Lower extremity joints angles	↑FL (11–18%SP)	NS	↑INV((-)200 m to (-)165 m, 78–100%SP)
Fuerst et al. (2018)	45°sidestep-cutting after a straight run	Shod	F	(-)100 m, IC	Ankle joint angles and movements	—	—	↓peak INV
Simpson et al. (2020a)	Forward jump and 45° crossover cut	—	F + S + T	IC to	Lower extremity joints movements	NS	↓ABDM(52–75%SP)	↑PLM(3–16%SP)
								↓EVM(39–81%SP)
Simpson et al. (2020b)	Forward jump and 45° crossover cut	—	F + S + T	IC to	Ankle joint angles	—	—	↑INT ROT (35–54%SP)

(-): before IC, (+): after IC, “-”: not measured in the study.

ABD, abduction; ABDM, abduction movement; EVM, eversion movement; F, frontal; FL, flexion; IC, initial contact; INV, inversion; INT ROT, internal rotation; NS, no significant differences between groups; PFM, plantarflexion movement; S, sagittal; SP, stance phase; T, transversal; TO, teo off.

### 3.5 Studies related to stop jump

There were three studies in all, with two including ankle kinematics, two involving knee kinematics (Lin et al., 2011; Terada et al., 2014a), and just one involving hip kinematics (Terada et al., 2014a), however, no study involving lower extremity kinetics was included. Table 3 presents a summary of the key characteristics of the included studies. There was no difference in hip flexion at maximal anterior tibial shear force (ATSF) between the CAI and control groups (Terada et al., 2014a). When compared to the control group, the CAI group had a smaller knee flexion angle at IC, peak ATSF, and 100 ms post-IC, with mean group differences of 6.28°, 6.70°, and 7.30°, respectively (Terada et al., 2014a; Terada et al., 2014b). The CAI group exhibited a greater ankle inversion than the control group 140 ms post-IC. Furthermore, the CAI group’s peak ankle eversion angle in the post-landing phase was lower than the control group’s (difference = 3.40°) (Lin et al., 2011). At peak ATSF, however, there was no difference between the groups in ankle dorsiflexion (Terada et al., 2014a).

**TABLE 3:** Summary of articles related to stop jump.

Authors	Task	Foot condition	Planes	Time frame	Variables	Hip	Knee	Ankle
Lin et al. (2011)	Vertical stop jump	Shod	F	(-)200 m to (+)200 m	Ankle angles	—	—	↑INV (0 m to (+)140 m)
								↓peak EV (0 to (+)200 m)
Terada et al. (2014a)	Vertical stop jump	Shod	S	Peak ATSF	Lower extremity joints angles	NA	↓FL	NA
Terada et al. (2014b)	Vertical stop jump	Shod	S	(-)100 m, IC, (+)100 m	Knee angels	—	↓FL (IC, (+)100 m)	—

(-): before IC, (+): after IC, “-“: not measured in the study.

ATSF, anterior tibial shear force; EV, eversion; F, frontal; FL, flexion; IC, initial contact; INV, inversion; NS, no significant differences between groups; S, sagittal.

### 3.6 Studies related to landing

In total, twenty-four studies were included. All of them involved ankle kinematics and kinetics, 15 studies involving hip kinematics or kinetics (Delahunt et al., 2006; Gribble and Robinson, 2009; Gribble and Robinson, 2010; De Ridder et al., 2015; Son et al., 2017; Herb et al., 2018; Kim et al., 2018; Kunugi et al., 2018; McCann et al., 2019; Kim et al., 2019; Hopkins et al., 2019; Lin et al., 2019; Moisan et al., 2020; Jeon et al., 2020; Watanabe et al., 2022), and 17 studies involving knee kinematics or kinetics (Caulfield and Garrett, 2002; Delahunt et al., 2006; Gribble and Robinson, 2009; Gribble and Robinson, 2010; De Ridder et al., 2015; Son et al., 2017; Herb et al., 2018; Kim et al., 2018; Li et al., 2018; Kunugi et al., 2018; McCann et al., 2019; Kim et al., 2019; Hopkins et al., 2019; Lin et al., 2019; Moisan et al., 2020; Jeon et al., 2020; Watanabe et al., 2022). Supplementary Table S2 provides a summary of the characteristics of all included studies.

The single-leg landing task was used in four studies (Li et al., 2018; Simpson et al., 2019b; Watabe et al., 2021; Watanabe et al., 2022). The mean platform height was 30 cm (range from 30 to 50 cm). As for the landing surfaces condition, two studies were flat (Watabe et al., 2021; Watanabe et al., 2022), one was a 20° inversion platform (Simpson et al., 2019b), and the other was a 5° inversion platform (Watabe et al., 2021). In studies using a flat landing surface (Watabe et al., 2021; Watanabe et al., 2022), no significant differences in hip angles or movements were identified between the CAI group and the control group (Watanabe et al., 2022). Peak knee flexion angle and peak ankle plantarflexion moment were bigger in the CAI group than in the control group in the 50 cm height platform condition, with a trend to be larger in the CAI group than in the control group in the 30 and 40 cm conditions. Furthermore, the CAI group had a greater peak ankle dorsiflexion angle than the control group at all heights. However, in the 30 cm height platform condition, Watanabe et al. (2022). (Watabe et al., 2021) showed no significant difference between groups for the greatest ankle inversion angle. There is no study on hip kinematics or kinetics when landing on an inverted surface; however, one study found that the CAI group had significantly higher knee values for flexion angle at IC (difference = 5.8°), peak flexion angle (difference = 15.7°), peak extension moment (difference = 0.27 Nm/kg), and peak internal rotation moment (difference = 0.12 Nm/kg) (Li et al., 2018). The CAI group had a 0.22 Nm/kg lower peak ankle eversion moment and a considerably larger maximum inversion angle than the control group (Li et al., 2018).

Five studies conducted single-leg jump landing tests with platform heights ranging from 35 to 46 cm (Caulfield and Garrett, 2002; Delahunt et al., 2006; Wright et al., 2016; Lin et al., 2019; Moisan et al., 2020). Only one study reported the distance from the platform to the center of the force plate was determined by the subject’s leg length (Lin et al., 2019), others were unclear. The landing surfaces in all five tests were flat, with only one including an unstable and 25° inversion platform (Moisan et al., 2020). When landing flat, compared to control, CAI patients exhibited a less externally rotated hip joint during a period from 200 to 55 ms pre-IC (Delahunt et al., 2006), as well as significantly larger hip flexion and hip adduction angle from initial contact to maximum knee flexion angle (Lin et al., 2019). Yet, Moisan et al. (Moisan et al., 2020) found no differences in hip angles across the groups. Furthermore, no difference in hip movements was identified across groups in any of the three studies. (Delahunt et al., 2006; Lin et al., 2019; Moisan et al., 2020). Caulfield et al. (Caulfield and Garrett, 2002) discovered that CAI had significantly more knee flexion than controls from 20 ms pre-IC to 60 ms post-IC, nevertheless, no difference was observed between groups for the remaining three studies (Delahunt et al., 2006; Lin et al., 2019; Moisan et al., 2020). Subjects with CAI demonstrated significantly greater ankle dorsiflexion from 10 ms pre-IC to 20 ms post-IC (Caulfield and Garrett, 2002), and less dorsiflexion from 90 to 200 ms post-IC (Delahunt et al., 2006). For the hindfoot, CAI displayed more dorsiflexion at IC (Wright et al., 2016). In the frontal plane, CAI individuals exhibited a more inverted ankle joint from 200 to 95 ms pre-IC (Delahunt et al., 2006), but a smaller ankle inversion angle and a significantly higher ankle eversion moment from initial contact to maximum knee flexion angle compared to controls (Lin et al., 2019). Moisan et al. (2020) on the other hand, found no differences in ankle joint angles and movement across groups. When landed on a laterally inclined surface, the CAI group showed greater knee extension moment during the landing phase compared to the control group and increased ankle dorsiflexion during the landing phase when landed on an unstable surface (Moisan et al., 2020).

Two studies performed a double-leg drop-vertical-jump task (DVJ) (Gehring et al., 2014; Herb et al., 2018). Participants in only one study were asked to perform a double-leg drop–vertical-jump task from a 30-cm box (Herb et al., 2018). In terms of hip angles and motions, there was no difference between CAI subjects and controls. From 95 to 200 ms post-IC, the CAI group had less knee flexion (difference = 8.23 ± 0.97°), however, there were no differences in knee joint moments between groups. The CAI group had more ankle inversion from 107 to 200 ms post-IC (difference = 4.01 ± 2.55°) and less plantar flexion from 11 to 71 ms post-IC (difference = 5.33° ± 2.02°). From 11 to 77 ms post-IC (difference = 0.17 ± 0.09 Nm/kg) and from 107 to 200 ms post-IC (difference = 0.23 ± 0.03 Nm/kg), the CAI group had a higher plantar-flexion moment (Herb et al., 2018). Another study asked participants to land at the platform with 24° inversion and 15° plantar flexion. They discovered that the maximum ankle inversion was much higher in CAI (difference = 5.5°) compared to the control group (Gehring et al., 2014).

Seven studies involved a double-leg take-off jump followed by a single-limb landing (Gribble and Robinson, 2009; Gribble and Robinson, 2010; Zhang et al., 2012; Kipp and Palmieri-Smith, 2012; De Ridder et al., 2015; McCann et al., 2019; Jeon et al., 2020). The platform height varied from 15 to 70 cm, and the distance between the platform and the force plate’s center was variable. All of the studies’ landing surfaces were flat. Two of them discovered that the CAI group had significantly greater hip joint flexion angles during landing than the controls (Jeon et al., 2020), and less hip abduction from 78 ms pre-IC to 34 ms post-IC (McCann et al., 2019). By contrast, the other three studies found no differences between groups in hip angles (Gribble and Robinson, 2009; Gribble and Robinson, 2010; De Ridder et al., 2015) or internal joint moments for hip joints between groups (Jeon et al., 2020). When compared to the CAI group, the control group produced significantly more knee flexion 100 ms pre-IC (difference = 94.29°) (Gribble and Robinson, 2010). The control group showed more knee flexion than the CAI group at IC (difference = 4.42°) (Gribble and Robinson, 2009). During the landing phase, the CAI group had more knee flexion than the control group (Jeon et al., 2020). Unlike, the other three studies found no significant difference in knee joint angles and movements between groups (De Ridder et al., 2015; McCann et al., 2019; Jeon et al., 2020). Impressively, none of the seven studies found any significant differences in ankle joint angles and moments across groups (Gribble and Robinson, 2009; Gribble and Robinson, 2010; Kipp and Palmieri-Smith, 2012; Zhang et al., 2012; De Ridder et al., 2015; McCann et al., 2019; Jeon et al., 2020).

**FIGURE 2:**
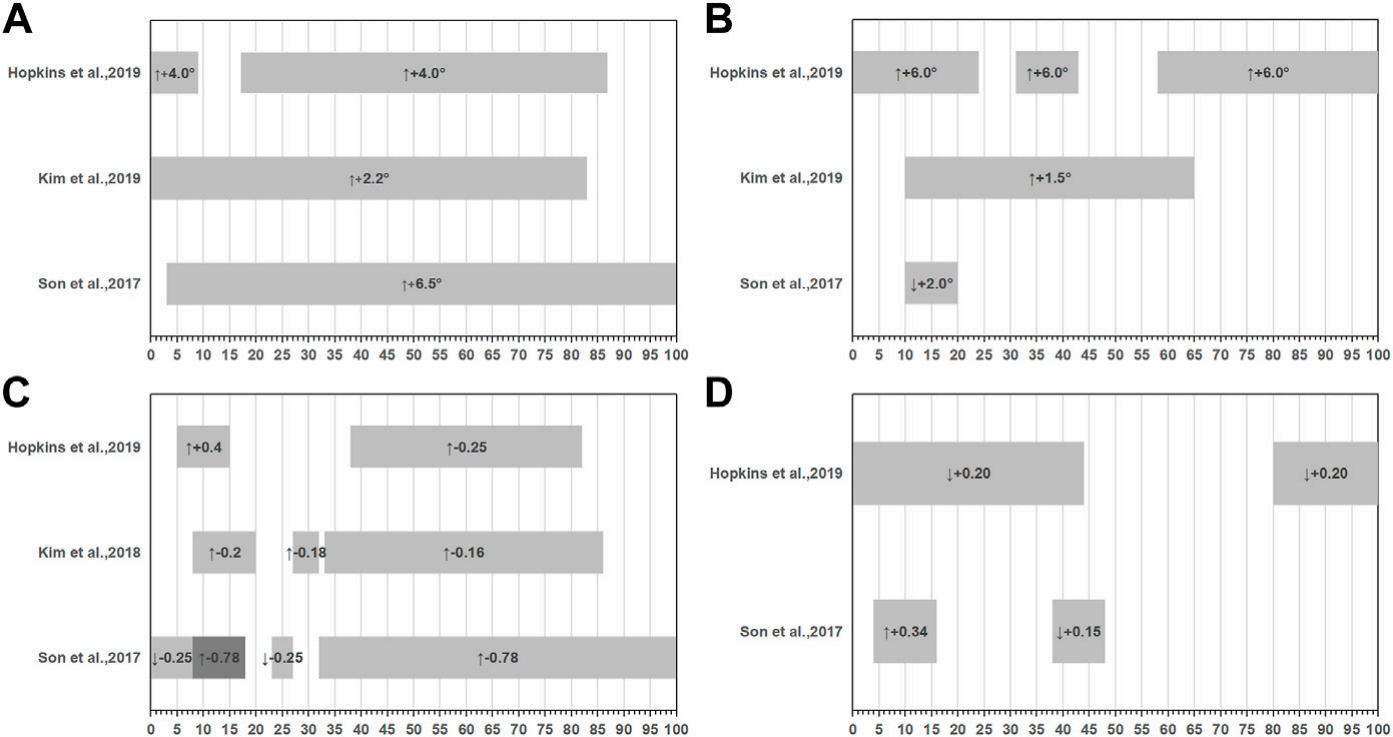
Summary of hip joint kinematics or kinetics during double-leg take-off jump with a single-limb landing and 90° cut task. **(A)** Sagittal hip angles, **(B)** Frontal hip angles, **(C)** Sagittal hip movements, **(D)** Frontal hip movements. (-): extension, (+): flexion or abduction, (↑): variables increased compared to the health group, (↓): variables decreased compared to the health group.

**FIGURE 3:**
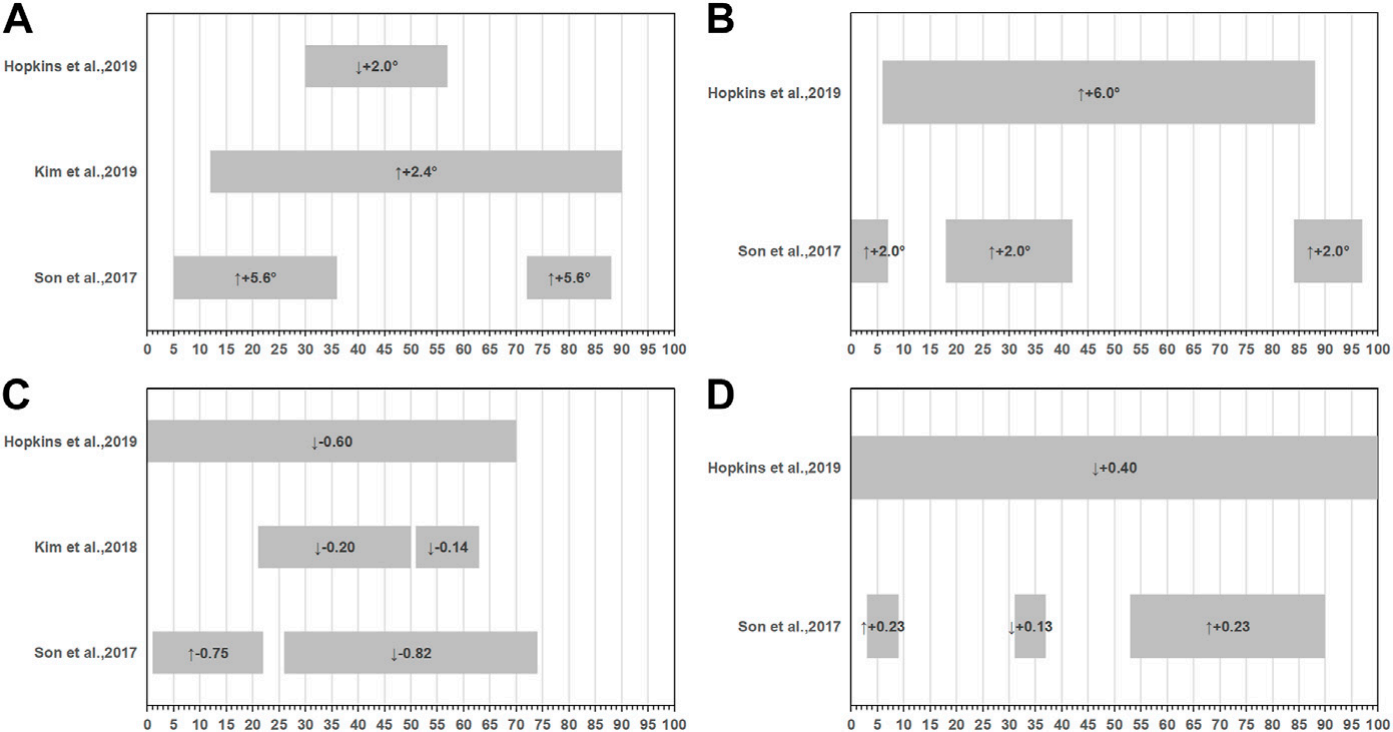
Summary of knee joint kinematics or kinetics during double-leg take-off jump with a single-limb landing and 90° cut task. **(A)** Sagittal knee angles, **(B)** Frontal knee angles, **(C)** Sagittal knee movements, **(D)** Frontal knee movements. (-): extension, (+): flexion or abduction, (↑): variables increased compared to the health group, (↓): variables decreased compared to the health group.

**FIGURE 4:**
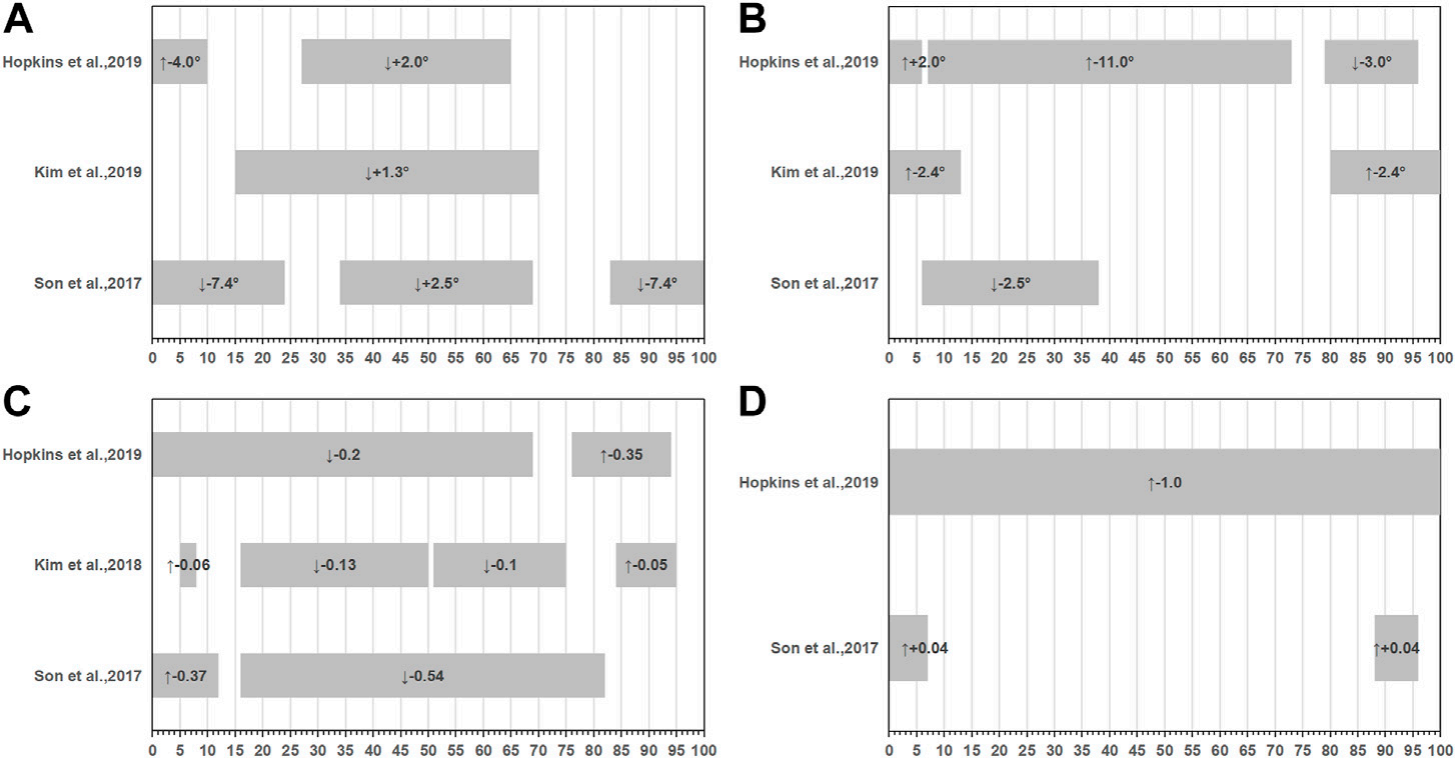
Summary of ankle joint kinematics or kinetics during double-leg take-off jump with a single-limb landing and 90° cut task. **(A)** Sagittal ankle angles, **(B)** Frontal ankle angles, **(C)** Sagittal ankle movements, **(D)** Frontal ankle movements. (-): plantarflexion or inversion, (+): dorsiflexion or eversion, (↑): variables increased compared to the health group, (↓): variables decreased compared to the health group.

Five studies conducted double-leg take-off jump with a single-limb landing and 90°cut task (Kipp and Palmieri-Smith, 2013; Son et al., 2017; Kim et al., 2018; Kim et al., 2019; Hopkins et al., 2019). In each of the five studies, the landing surface was flat. Ground contact was divided into two parts during this task: the landing phase, which lasted from first ground contact to peak joint flexion, approximately 0%–50% of stance, and the side-cutting phase, which lasted from the end of the landing through takeoff, approximately 51%–100% of stance. In the case of the hip joint, relative to controls, CAI patients decency with more hip flexion angle (Son et al., 2017; Kim et al., 2019; Hopkins et al., 2019), more hip abduction (Son et al., 2017; Kim et al., 2019; Hopkins et al., 2019), higher hip extension moment (Son et al., 2017; Kim et al., 2018; Hopkins et al., 2019), and less hip abduction moment (Son et al., 2017; Hopkins et al., 2019) (Figure 2). In terms of the knee joint, CAI patients displayed a tendency with more knee flexion (Son et al., 2017; Kim et al., 2019; Hopkins et al., 2019), more knee abduction angle (Son et al., 2017; Hopkins et al., 2019), less knee extension moment (Son et al., 2017; Kim et al., 2018; Hopkins et al., 2019), and less knee abduction (Son et al., 2017; Hopkins et al., 2019) moments relative to controls (Figure 3). As for ankle joint, results are inconsistent across studies, Figure 4 summarizes results from each study.

Only a single study performed a double-leg take-off jump 45° anterolateral from a 30 cm height platform with a single-limb landing at the flat and vertical-jump task. When compared to the control group, the CAI group had significantly less hip adduction (difference = 3.99°) from 70 to 120 ms post-IC, less knee flexion (difference = 7.33°) from 120 to 190 ms post-IC, less knee external rotation (difference = 4.14°) from 300 to 250 ms pre-IC, and less ankle dorsiflexion (difference = 12.83°) from 10 ms pre-IC to 300 ms post-IC (Kunugi et al., 2018).

## 4 DISCUSSION

This study was designed to systematically review and evaluate the effects of CAI on lower extremity joint angles and moments during side-cut, stop jump, and landing tasks. This study found that the biomechanical changes of the proximal lower extremity joints caused by CAI may increase the risk of ACL injury during high-risk movements of ACL injury, such as side-cut, stop jumps, and landing.

### 4.1 Changes in ankle biomechanics

The greater trend observed in the ankle dorsiflexion position in the CAI subjects during the early phase after IC is a trend towards greater protection of the lateral ligamentous complex because in the dorsiflexion position, the ligaments is unlikely to be stretched. During the landing procedure, subjects may have been subconsciously striving to reduce the consequences of ground impact forces on their ligaments. In a recent systematic review, it was hypothesized that increased ankle dorsiflexion during jump landing for people with CAI could be due to centrally mediated motor program changes caused by the unstable ankle, which would place the talocrural joint in a tightly packed position to protect the lateral ankle ligaments from excessive inversion (Simpson et al., 2019b).

However, CAI subjects were not as efficient as control group subjects in obtaining the closed-packed dorsiflexed posture of the ankle joint during the late phase after IC. This could indicate a restriction in the posterior talar glide. Limited dorsiflexion range of motion has been identified as a factor in the development of lateral ankle joint injuries (Tabrizi et al., 2000). The decrease in peroneal longus activity before IC leaves the ankle joint in a vulnerable position (i.e., a more inverted position) and induces a hyper inversion injury in CAI participants (Delahunt et al., 2006). Since the ankle is in a less dorsiflexion position it is unable to absorb forces as well and greater forces are transmitted to the knee, increasing the risk factors that can lead to non-contact knee injuries (Theisen and Day, 2019).

CAI patients had a more inverted foot position after IC, which was attributed to a lack of ankle proprioception (Lin et al., 2011) and diminished peroneus muscle activity (Delahunt et al., 2006). Because of subtalar instability, this higher inversion ankle position at IC has also been linked to an increased risk of ankle roll-over and sprain injury (Yamamoto et al., 1998). The greater the ankle inversion angle, the greater the risk of recurring lateral ankle sprain (Lin et al., 2011). When the lateral ankle is loaded during IC, reduced ankle eversion moments indicate that the lateral ankle muscle is unable to control frontal plane movement eccentrically (Son et al., 2017; Kim et al., 2018). This could also cause excessive inversion of the ankle complex, resulting in a recurrent lateral ankle sprain.

### 4.2 Changes in knee biomechanics

Participants with CAI had a small flexion angle of the knee joint (Terada et al., 2014b) after IC. A straightened knee has less potential energy decay and could indicate a lack of absorption capabilities (Herb et al., 2018). However, there may be a compensatory strategy that allows CAI participants to absorb kinetic energy from their bodies. When the ankle and knee joints land on the ground, doing work on the eccentric part of the sagittal frontal plane will help to reduce the body’s kinetic energy in the vertical direction (Norcross et al., 2013). Increased knee extension at impact may provide the ground reaction forces more time to dissipate and control. These findings of changes in knee biomechanics following ground impact suggest that the presence of CAI may alter the distal-to-proximal connection that provides an efficient and effective system for transmitting forces up the kinetic chain (Dejong et al., 2020). Decreased knee flexion angle can result in insufficient energy attenuation capabilities of the knee, resulting in the knee joint receiving large compressive impact forces, increasing the stress on the ATSF and the load on the ACL (Chappell et al., 2005; Norcross et al., 2010). Nevertheless, those biomechanical changes in the knee joint are related to the mechanism of ACL injury (Fleming et al., 2001) and may increase the risk of ACL injury.

### 4.3 Changes in hip biomechanics

Due to induced sensorimotor loss at the ankle, CAI participants demonstrate increased hip joint dependency to maintain balance and stability during those three sports maneuvers (Horak et al., 1990). Subjects with CAI may attempt to adjust to a position relative to their low center of mass for dynamic stability, primarily utilizing hip flexion (Koshino et al., 2014). CAI may be able to obtain enough balance to adequately stabilize themselves in the sagittal plane by increasing hip flexion motion to mitigate the effects of GRF (Lin et al., 2019). Increased hip flexion angles can help the eccentrically controlled hip extensors absorb or dissipate the impact more effectively over time. Participants with CAI may attempt to land safely with more flexed hip positions, protecting the unstable ankle from the high-impact landing (Son et al., 2017). It may be easier and safer for CAI patients to have their femurs more vertically upright (less abducted), which may help stabilize the downward motion of their center of body mass in the sagittal plane. A compensatory load redistribution strategy from the unstable distal (e.g., ankle) to proximal (e.g., hip) joints, indicates that the hip joint in the sagittal plane may play an important role in sports maneuvers (Son et al., 2017). However, CAI patients displayed higher hip adduction over the majority of the task’s stance phase, possibly due to decreased frontal plane hip joint stability (Son et al., 2017). Increased hip abduction may prevent excessive ankle inversion motion (Koshino et al., 2014). People with CAI may have developed a strategy to alter frontal plane hip kinematics to compensate for ankle instability during the task (Dejong et al., 2020).

### 4.4 Reason for inconsistent results

Although this systematic review discovered that CAI participants demonstrate a changed movement strategy during sports actions involving cutting, stopping jumping, and landing, these changes are not limited to the ankle joint, but rather manifest farther up the kinematic chain (e.g., knee, and hip). The clinical variability among studies was found to be significant in this systematic review. Disparities of this nature might be classified into five categories. The first category is concerned with the various authors’ definitions of CAI. Different inclusion criteria were applied in different studies, resulting in a non-homogeneous population. As a result, comparing data from different studies is challenging. The International Ankle Consortium established selection criteria guidelines to follow when researchers assess participants with CAI in 2013 (Gribble et al., 2013). However, it should be noted that several of the studies included in this systematic review (8/32) were published before these principles were widely recognized. The second category refers to the sports level of participants. Half of the included studies (15/32) had recreational physical activity participants as subjects, while others (6/32) had athletes as subjects. However, some research classified recreational exercise as “at least 20 min of strenuous activity, three or more days per week” (Terada et al., 2014a; Terada et al., 2014b) while others were “at least 30 min of exercise three times per week.” (Gribble and Robinson, 2009). The third category refers to discrepancies in the experimental tasks, such as the height of the platform and the surface landing condition. The fourth category refers to the food conditions in which the participants performed the experimental tasks. Some studies measured kinematics and kinetics data while the subjects were wearing shoes, while others measured them while they were barefoot. The fifth category is related to various data processing methods. Lower limb kinematics or kinetics parameters were calculated at initial contact or point of maximum vertical ground reaction force in some studies, while some studies used the one-dimensional statistical parametric mapping (SPM) analysis. The external validity of the outcomes of this systematic review is compromised by all of these variations. As a result, there was no way to pool the data and perform a meta-analysis.

## 5 Conclusion

The changed angle and movements of the knee and hip found in CAI participants during sports maneuvers involving side cutting, stop jumping, and landing might be a result of central nervous system modifications following a peripheral ankle joint injury (Ward et al., 2015). Because of the mechanical advantages of the proximal joints, participants with CAI may evolve an altered movement strategy to redistribute the force from the unstable distal joint (e.g., ankle) to the more stable proximal joints (e.g., knee and hip). This shift in movement pattern, on the other hand, transmitting greater forces to the knee, which increases the load on ACL, may be linked to an increased risk of ACL injuries.

## Data Availability

The original contributions presented in the study are included in the article/Supplementary Material, further inquiries can be directed to the corresponding authors.
