# Supplementary material for: Chronic ankle instability modifies proximal lower extremity biomechanics during sports maneuvers that may increase the risk of ACL injury: A systematic review
Source: Front Physiol. 2022 Oct 18;13:1036267. doi: 10.3389/fphys.2022.1036267 (PMC9623000; doi:10.3389/fphys.2022.1036267)
Supplement: Supplementary file 2 [file Table1.DOC]

SUPPLEMENTARY TABLE 1 Risk of bias in retrospective studies using modified Newcastle-Ottawa scale

| Author | Selection | | | | Comparability†* | Outcome | | | Quality score |
| --- | --- | --- | --- | --- | --- | --- | --- | --- | --- |
|  | Definition of CAI groups | Definition of control groups | Representative of CAI groups | Representative of control groups |  | Clear test method | Uniform test methods | Non-response rate |  |
| Caulfield et al.,2002 | Yes | Yes | Yes | Yes | 1,5 | Yes | Yes | Unclear | 7 |
| Delahunt et al.,2006 | Yes | Yes | Yes | Yes | 3,4 | Yes | Yes | Unclear | 7 |
| Gribble et al.,2009 | Yes | Yes | Yes | Yes | All | Yes | Yes | Unclear | 8 |
| Gribble et al.,2010 | Yes | Yes | Yes | Yes | All | Yes | Yes | Unclear | 8 |
| Lin et al.,2011 | Yes | NA | Yes | Yes | 1,2,3,4,5 | Yes | Yes | Unclear | 6 |
| Kipp et al.,2012 | Yes | Yes | Yes | Yes | 1,3,4 | Yes | Yes | Unclear | 7 |
| Zhang et al.,2012 | Yes | Yes | Yes | Yes | 3,4 | Yes | Yes | Unclear | 7 |
| Kipp et al.,2013 | Yes | NA | Yes | Yes | 1,2,3,4,5 | Yes | Yes | Unclear | 6 |
| Terada et al.,2014a | Yes | Yes | Yes | Yes | All | Yes | Yes | Unclear | 8 |
| Terada et al.,2014b | Yes | Yes | Yes | Yes | 1,2,3,4,5 | Yes | Yes | Unclear | 7 |
| Koshino et al.,2014 | Yes | Yes | Yes | Yes | All | Yes | Yes | Unclear | 8 |
| Gehring et al.,2014 | Yes | Yes | Yes | Yes | NA | Yes | Yes | Unclear | 6 |
| De Ridder et al.,2015 | Yes | Yes | Yes | Yes | 1,2,3,4,5 | Yes | Yes | Unclear | 7 |
| Koshino et al.,2016 | Yes | Yes | Yes | Yes | All | Yes | Yes | Unclear | 8 |
| Wright et al.,2016 | Yes | Yes | Yes | Yes | 1,2,3,4,6 | Yes | Yes | Unclear | 7 |
| Son et al.,2017 | Yes | Yes | Yes | Yes | 1,3,4,5,6 | Yes | Yes | Unclear | 7 |
| Fuerst et al.,2018 | Yes | Yes | Yes | Yes | 1,2,3,4 | Yes | Yes | Unclear | 7 |
| Herb et al.,2018 | Yes | Yes | Yes | Yes | 1,3,4,5,6 | Yes | Yes | Unclear | 7 |
| Kim et al.,2018 | Yes | Yes | Yes | Yes | All | Yes | Yes | Unclear | 8 |
| Li et al.,2018 | Yes | Yes | Yes | Yes | All | Yes | Yes | Unclear | 8 |
| Kunugi et al.,2018 | Yes | Yes | Yes | Yes | 1,2,3,4 | Yes | Yes | Unclear | 7 |
| McCann et al.,2019 | Yes | Yes | Yes | Yes | 2,3,4 | Yes | Yes | Unclear | 7 |
| Kim et al.,2019 | Yes | Yes | Yes | Yes | All | Yes | Yes | Unclear | 8 |
| Hopkins et al.,2019 | Yes | Yes | Yes | Yes | 5 | Yes | Yes | Unclear | 6 |
| Lin et al.,2019 | Yes | Yes | Yes | Yes | All | Yes | Yes | Unclear | 8 |
| Simpson et al.,2019 | Yes | NA | Yes | Yes | 1,3,4, | Yes | Yes | Unclear | 6 |
| Moisan et al.,2020 | Yes | NA | Yes | Yes | 1,2,3,4 | Yes | Yes | Unclear | 6 |
| Jeon et al.,2020 | Yes | NA | Yes | Yes | 1,2,3,4,6 | Yes | Yes | Unclear | 6 |
| Simpson et al.,2020a | Yes | NA | Yes | Yes | 1,2,3,4,6 | Yes | Yes | Unclear | 6 |
| Simpson et al.,2020b | Yes | NA | Yes | Yes | 1,2,3,4,6 | Yes | Yes | Unclear | 6 |
| Watabe et al.,2021 | Yes | Yes | Yes | Yes | 1,2,3,4,6 | Yes | Yes | Unclear | 7 |
| Watanabe et al.,2022 | Yes | Yes | Yes | Yes | All | Yes | Yes | Unclear | 8 |

†Comparability variables: 1=gender; 2=age; 3=hight; 4=weight; 5=sports level; 6=dominance side

*If all characteristics were comparable, 2 points; if two or three characteristics were comparable, 1 point; otherwise, no point
